# Targeting metabolic pathways: a novel therapeutic direction for type 2 diabetes

**DOI:** 10.3389/fcimb.2023.1218326

**Published:** 2023-08-02

**Authors:** Zhihui Song, An Yan, Zehui Guo, Yuhang Zhang, Tao Wen, Zhenzhen Li, Zhihua Yang, Rui Chen, Yi Wang

**Affiliations:** ^1^ State Key Laboratory of Component-based Chinese Medicine, Tianjin University of Traditional Chinese Medicine, Tianjin, China; ^2^ Institute of Traditional Chinese Medicine, Tianjin University of Traditional Chinese Medicine, Tianjin, China; ^3^ Tianjin University of Traditional Chinese Medicine, Tianjin Academy of Traditional Chinese Medicine Affiliated Hospital, Tianjin, China; ^4^ College of Traditional Chinese Medicine, Tianjin University of Traditional Chinese Medicine, Tianjin, China

**Keywords:** type 2 diabetes, metabolites, gut microbiota, dopamine, estradiol

## Abstract

**Background:**

Type 2 diabetes mellitus (T2DM) is a prevalent metabolic disease that causes multi-organ complications, seriously affecting patients’ quality of life and survival. Understanding its pathogenesis remains challenging, with current clinical treatment regimens often proving ineffective.

**Methods:**

In this study, we established a mouse model of T2DM and employed 16s rDNA sequencing to detect changes in the species and structure of gut flora. Additionally, we used UPLC-Q-TOF-MS to identify changes in urinary metabolites of T2DM mice, analyzed differential metabolites and constructed differential metabolic pathways. Finally, we used Pearman correlation analysis to investigate the relationship between intestinal flora and differential metabolites in T2DM mice, aiming to elucidate the pathogenesis of T2DM and provide an experimental basis for its clinical treatment.

**Results:**

Our findings revealed a reduction in both the species diversity and abundance of intestinal flora in T2DM mice, with significantly decreased levels of beneficial bacteria such as Lactobacillus and significantly increased levels of harmful bacteria such as Helicobacter pylori. Urinary metabolomics results identified 31 differential metabolites between T2DM and control mice, including Phosphatidylcholine, CDP-ethanolamine and Leukotriene A4, which may be closely associated with the glycerophospholipid and arachidonic acid pathways. Pearman correlation analysis showed a strong correlation between dopamine and gonadal, estradiol and gut microbiota, may be a novel direction underlying T2DM.

**Conclusion:**

In conclusion, our study suggests that alterations in gut microbiota and urinary metabolites are characteristic features of T2DM in mice. Furthermore, a strong correlation between dopamine, estradiol and gut microbiota, may be a novel direction underlying T2DM, the aim is to provide new ideas for clinical treatment and basic research.

## Graphical Abstract

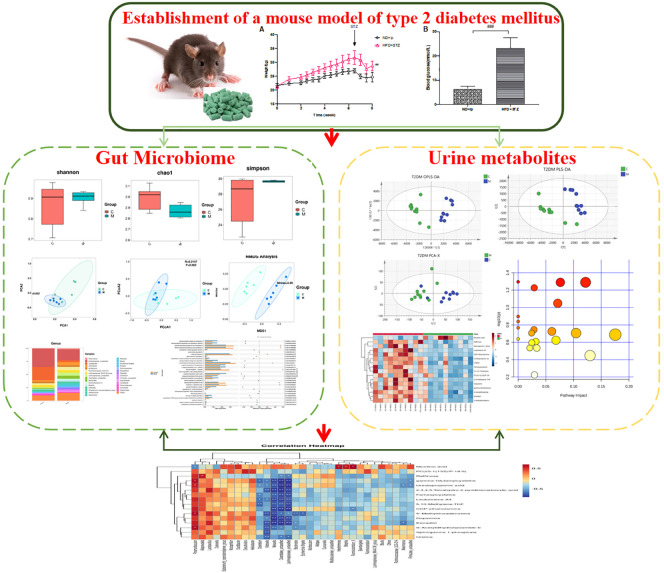

## 1 Introduction

Type 2 diabetes (T2DM) is a chronic metabolic disease that primarily manifests as hyperglycemia due to impaired insulin secretion and/or action resulting from various etiologies. New estimates published in The Lancet indicate that more than 1.31 billion people could be living with diabetes by 2050 worldwide (The, 2023). The pathogenesis of T2DM remains unclear, with current research implicating genetic and environmental factors, as well as defects in insulin secretion, inflammation, and metabolism. The long-term metabolic abnormalities associated with T2DM can lead to multi-system complications, including cardiovascular disease, visual impairment, neurological disease, renal disease, and diabetic foot, ultimately resulting in increased morbidity and mortality rates (Ussher and Drucker, 2023). T2DM can have a profound negative impact on patients’ quality of life, impairing their ability to work and participate in daily activities. This not only increases the medical burden on society but also places a significant financial strain on families affected by the disease (Li et al., 2023). In conclusion, the high and increasing prevalence of T2DM, driven by changes in global economies, social structures, and lifestyles, represents a major challenge for global health and economies.

The gut microbiome is a crucial component of the body’s microbial community, playing a critical role in regulating numerous bodily functions, including immunity, metabolism, and nutrition. Emerging evidence suggests that imbalances in the gut microbiome may be closely linked to the pathogenesis of T2DM (Li et al., 2020; Guan et al., 2023). Studies have shown a significant association between changes in the gut microbiota and the development of T2DM. The gut microbiota plays an important role in regulating human metabolism and immune function, but patients with T2DM have distinct alterations in the total number, diversity and composition of gut microbial communities compared to healthy individuals (Polidori et al., 2022). Studies have shown that changes in the gut microbiome of T2DM include not only changes in the number, composition and diversity of the microbiota, but also changes in the levels of metabolites in the gut microbiome, which can inhibit insulin release and lead to the development of T2DM (Bao et al., 2023). Second, changes in the gut microbiome are relevant to the treatment of T2DM: in the treatment of patients with T2DM, the use of probiotics and prebiotics can regulate the balance of the gut microbiome, thereby improving insulin resistance and blood glucose levels in patients with T2DM (Paul et al., 2022; Chen et al., 2023; Dixon et al., 2023). Again, the mechanism of action of intestinal flora changes in T2DM: intestinal flora can influence the development and progression of T2DM through a variety of mechanisms, including altering nutrient metabolism, affecting the intestinal barrier and immune system, and influencing neurological regulation (Tawulie et al., 2023). In addition, changes in the levels of gut flora metabolites, such as short chain fatty acids (SCFAs), and metabolites, such as secondary bile acids, can affect the host’s energy metabolism and insulin sensitivity, thereby influencing the onset and progression of T2DM (Gu et al., 2023). SCFAs exert beneficial metabolic effects by acting as energy substrates in the TCA cycle, regulating hormones involved in satiety regulation and insulin secretion, and regulating immune cells and microglia (Luo et al., 2022). In conclusion, the onset and development of T2DM is closely linked to changes in the gut microbiome, and the balance of the gut microbiome can be adjusted by altering the composition of the gut microbiome and regulating the levels of metabolites, which can help prevent and treat T2DM. At the same time, by studying the changes in the gut flora and their mechanisms in depth, new ideas and methods for the treatment and prevention of T2DM can be provided, which is expected to provide better protection for the health of T2DM patients.

Urine metabolomics is a technique for studying changes in the composition and concentration of metabolites in urine, using high-throughput mass spectrometry analysis to quantify and identify thousands of compounds in urine, including sugars, fats and amino acids (Zhang et al., 2016). Previous studies have shown that urinary levels of sugar metabolites in type 2 diabetic are usually significantly elevated, for example, the levels of monosaccharides are increased, reflecting the impaired glucose metabolism caused by insulin resistance and impaired pancreatic β-cell function in T2DM (Zhao et al., 2020; Wang et al., 2021; Li et al., 2022). Other studies have shown that the levels of amino acid metabolites in the urine of T2DM are also affected, A branched-chain amino acid metabolite drives vascular fatty acid transport and causes insulin resistance (Giesbertz and Daniel, 2016; Jang et al., 2016). Therefore, a systematic study on the changes of urinary metabolites in T2DM mice can reveal the pathophysiological processes and possible targets of T2DM, and provide new ideas and methods for the diagnosis, treatment and prognosis of T2DM.

In this study, a type 2 diabetic animal model was established by intraperitoneal injection of streptozotocin (STZ) after 7 weeks of high-fat, high-sugar diet, and changes in the structure and species of mouse gut flora were detected by 16sRNA sequencing, and changes in urinary metabolites in type 2 diabetic mice were detected by UPLC-Q-TOF-MS technique to analyse the differential metabolites and construct differential metabolic pathways, Finally, the application of Pearman correlation analysis was used to explore the relationship between gut flora species and urinary metabolites in type 2 diabetic mice, to reveal possible targets of T2DM, and to provide new ideas and methods for the diagnosis, treatment and prognosis of T2DM (**Graphical Abstract**).

## 2 Methods

### 2.1 Animals

Eight-week-old male C57BL/6J mice were purchased from SiPeiFu (Beijing Biotechnology). The mice were raised in the Animal Center of Tianjin University of Traditional Chinese Medicine (TCM–LAEC2021037). All the experimental protocols were conducted in accordance with the guidelines approved by the Animal Care Committee of Tianjin University of Traditional Chinese Medicine and Animal Ethical Committee of Tianjin University of Traditional Chinese Medicine (TCM–LAEC2021037).

### 2.2 Experiment protocol

Animals were allowed to acclimatize for a week and randomly divided into 2 groups:

the normal control group (Control: n=10, normal-chow-diet) and the model group (Model: n=10, 60% high-fat-diet-fed (HFD). The diets of the model group of mice were furnished by Medicience, Professional For Lab Animal Diet (MD12033). Mice in the model group were routinely supplied with a high-fat diet for 7 weeks prior to three consecutive intraperitoneal injections of 80 mg/kg/day of streptomycin (STZ) citrate buffer (pH 4.5). The blank group was injected with an equal volume of sodium citrate buffer. The mice were fasted overnight before receiving STZ injection. The mice’s fasting blood glucose levels were measured 72 hours after the last injection. The mice with FBG ≥ 16.7mmol/L were considered as diabetic mice.

### 2.3 The method of testing fasting blood sugar

After a 12 hour fast, tail vein blood samples of all mice were collected to determine the fasting blood glucose (FBG) level using a glucose meter and glucose test papers (SanRuo, China).

### 2.4 Analysis of gut microbiota

#### 2.4.1 DNA extractions

DNA from different samples was extracted using the CTAB according to manufacturer ‘s instructions. The reagent which was designed to uncover DNA from trace amounts of sample has been shown to be effective for the preparation of DNA of most bacteria. Nuclear-free water was used for blank. The total DNA was eluted in 50 μL of Elution buffer and stored at -80°C until measurement in the PCR by LC-Bio Technology Co., Ltd, Hang Zhou, Zhejiang Province, China.

#### 2.4.2 PCR amplification and 16S rDNA sequencing

The 5’ ends of the primers were tagged with specific barcodes per sample and sequencing universal primers. PCR amplification was performed in a total volume of 25 μL reaction mixture containing 25 ng of template DNA, 12.5 μL PCR Premix, 2.5 μL of each primer, and PCR-grade water to adjust the volume. The PCR conditions to amplify the prokaryotic 16S fragments consisted of an initial denaturation at 98°C for 30 seconds; 32cycles of denaturation at 98°C for 10 seconds, annealing at 54°C for 30 seconds, and extension at 72°C for 45 seconds; and then final extension at 72°C for 10 minutes. The PCR products were confirmed with 2% agarose gel electrophoresis. Throughout the DNA extraction process, ultrapure water, instead of a sample solution, was used to exclude the possibility of false-positive PCR results as a negative control. The PCR products were purified by AMPure XT beads (Beckman Coulter Genomics, Danvers, MA, USA) and quantified by Qubit (Invitrogen, USA). The amplicon pools were prepared for sequencing and the size and quantity of the amplicon library were assessed on Agilent 2100 Bioanalyzer (Agilent, USA) and with the Library Quantification Kit for Illumina (Kapa Biosciences, Woburn, MA, USA), respectively. The libraries were sequenced on NovaSeq PE250 platform.

#### 2.4.3 Data analysis

Samples were sequenced on an Illumina NovaSeq platform according to the manufacturer’s recommendations, provided by LC-Bio. Paired-end reads was assigned to samples based on their unique barcode and truncated by cutting off the barcode and primer sequence. Paired-end reads were merged using FLASH. Quality filtering on the raw reads were performed under specific filtering conditions to obtain the high-quality clean tags according to the fqtrim (v0.94). Chimeric sequences were filtered using Vsearch software (v2.3.4). After dereplication using DADA2, we obtained feature table and feature sequence. Alpha diversity and beta diversity were calculated by normalized to the same sequences randomly. Then according to SILVA(release 138) classifier, feature abundance was normalized using relative abundance of each sample. Alpha diversity is applied in analyzing complexity of species diversity for a sample through 5 indices, including Chao1, observed species, Goods coverage, Shannon, Simpson, and all this indices in our samples were calculated with QIIME2. Beta diversity were calculated by QIIME2, the graphs were drew by R package. Blast was used for sequence alignment, and the feature sequences were annotated with SILVA database for each representative sequence. Other diagrams were implemented using the R package (v3.5.2).

### 2.5 Urine metabolomic analysis

#### 2.5.1 Urine sample preparation

Urine was collected from mice (n=10) for 24 h at week 12 after drug administration, centrifuged at 1000 rpm/min for 10 min at 4°C to remove particulate contaminants, then the supernatant was centrifuged at 2500 rpm/min for 10 min and the supernatant was stored at -80°C for measurement.

#### 2.5.2 Chromatographic and mass spectrometric conditions

Chromatographic conditions: Column: ACQUITY UPLC BEH C18 (2.1 x 100 mm, 1.7 μm), flow rate: 0.3 mL/min, column temperature: 40°C, injection volume: 5 μL,

Mass spectrometric conditions: Mass spectrometric analysis was performed using an electrospray ionisation (ESI) source in both positive and negative ion modes. High purity N2 was used as the auxiliary spray ionisation and desolubilising gas, drying gas flow rate 10 mL/min, N2 temperature: 350°C, atomising gas pressure: 310 kPa, desolventizing nitrogen flow rate: 600 L/h, cone hole blowback nitrogen: 50 L/h. Capillary ionisation voltage: 2.1 kV, quadrupole scan range: m/z 50-1000.

#### 2.5.3 Methodological study

①Check the accuracy of the instrument: Take the same QC sample solution, inject the sample 6 times in succession, randomly select 20 peaks and calculate the RSD values of the peak area and retention time of the 20 peaks.

②Method accuracy test: 6 QC samples were prepared in parallel and analysed by sequential injections, 20 peaks were randomly selected.

#### 2.5.4 Metabolomics data processing and analysis

①Using UPLC-Q-TOF-MS technology, the raw data were pre-processed by MassLynxSCN 633 software. After pre-processing by the software, peak identification, peak alignment and peak filtering of the raw data can be performed automatically, and finally the retention time (Rt), mass-to-charge ratio (m/z) and spectral peak area were output to find out the potential discriminating variables.

②Unsupervised principal component analysis (PCA) was performed by multivariate statistical analysis of the data obtained from the serum metabolic profiles of each group of mice using SIMCA software. PCA was used to observe the distribution of metabolites and the overall differences between the groups of samples, while partial least squares discriminant analysis (PLS-DA) and orthogonal least squares discriminant analysis (OPLS-DA) allowed supervised downscaling of data patterns.

③Variable importance analysis (VIP) can be obtained in the OPLS-DA model by screening metabolites with VIP > 1 as differential metabolites. Independent samples t-tests were performed by comparing groups two by two, and compounds with P< 0.05 were used as significantly different end metabolites.

④The m/z values of the screened metabolites were used to identify potential metabolic markers in the HMDB database (http://www.hmdb.ca/), with H+, Na+ and K+ selected in positive ion mode. The metabolite name, chemical formula and parts per million (ppm) were recorded, and metabolites with |ppm| > 15 were excluded.

⑤To further characterise the trend of differential metabolites between groups, the normalised data were heat mapped. Enrichment of differential metabolite pathways by KEGG database (http://www.genome.jp/kegg/).

### 2.6 Statistical analysis

All data are presented as mean ± standard deviation (SD). P<0.05 was considered statistically significant. Application of Pearman correlation analysis to examine the association between urinary metabolites and gut microbiota.

## 3 Results

### 3.1 Reproduction of the T2DM model

**Figure 1:**
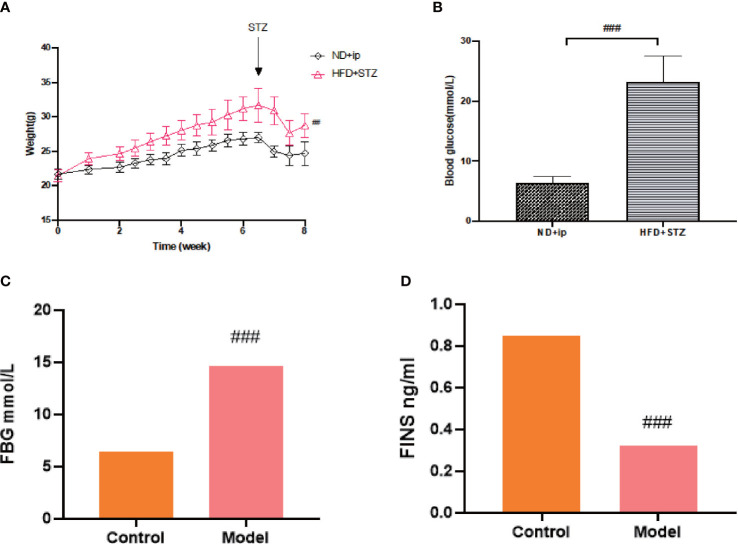
Establishment of T2DM Model. **(A)** Changes in body weight of mice during 8 weeks of moulding, ND+ip for control group, HFD+STZ for model group. **(B)** Comparison of blood glucose levels of the experimental groups after 8 weeks of moulding (p less than 0.001), ND+ip for control group, HFD+STZ for model group. **(C)** FBG in T2DM mice; **(D)** FINS levels in T2DM mice. ### p<0.001.

After 7 weeks of feeding the mice with a high fat and high sugar diet, fasting blood glucose levels were measured 72 h after the last intraperitoneal injection of 80 mg/kg STZ, and FBG≥16.7 mmol/L was considered a successful model. Compared to the control group, the model group showed a statistically significant increase in blood glucose (**Figure 1B**), and compared to the control group, the model group showed an increase in body weight, with a significant difference at week 8 (**Figure 1A**).

T2DM is a chronic metabolic disease, so we did not collect urine and faeces etc. immediately after judging that it caused a T2DM model, on which basis we continued feeding for 8 weeks and tested fasting blood glucose and insulin levels before proving a chronic T2DM model. (**Figures 1C, D**)

### 3.2 Changes in the gut microbiome of type 2 diabetic mice

#### 3.2.1 T2DM affected the community diversity and richness of the gut microbiome

**Figure 2:**
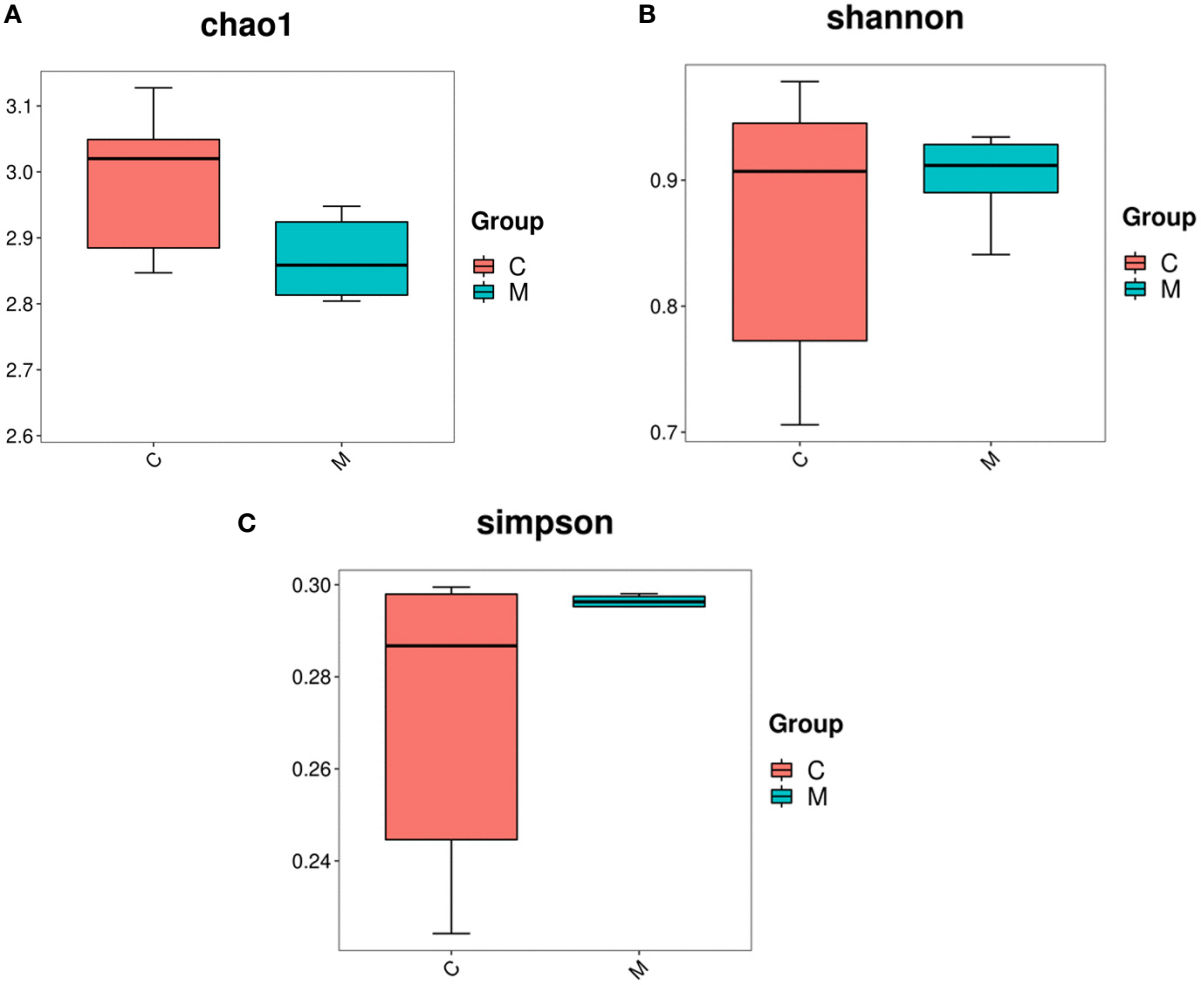
Diversity and abundance of gut microbiota in T2DM mice. 16S rDNA gene sequencing was used to evaluate the alterations in the gut microbiome induced by T2DM. Community richness and diversity were measured by the alpha diversity metrics. **(A)** Estimation of the number of species contained in the community according to the Chao1 index, C for the control group and M for the model group; **(B)** comparison of the difference in diversity between the two groups according to the Shannon index, the higher the Shannon index, the higher the uncertainty. Higher uncertainty indicates more unknowns in this community, i.e. high diversity, C for the control group and M for the model group; **(C)** the difference in richness between the two groups compared by the Simpson index, C for the control group and M for the model group.

16S rDNA gene sequencing was used to evaluate the alterations in the gut microbiome induced by T2DM. Community richness and diversity were measured by the alpha diversity metrics. The richness estimator Chao1 showed significant difference among Control and Model (**Figure 2A**). The Simpson’s and Shannon’s index of diversity revealed that Model group exhibited increased gut microbiome diversity compared to Control group (**Figures 2B, C**).

#### 3.2.2 T2DM changed the gut microbiome composition

**Figure 3:**
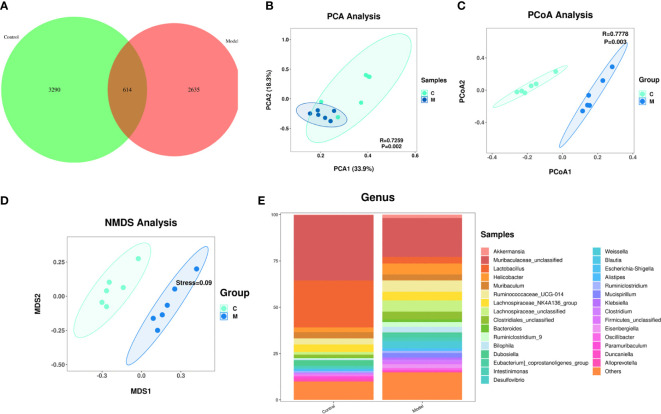
Changes in the composition of the gut microbiome of type 2 diabetic mice. **(A)** Change in the number of OTUs, control group in green, model group in pink; **(B)** Graph of the PCA analysis, control group in green, model group in blue; **(C)** Graph of the PcoA analysis, control group in green, model group in blue; **(D)** Graph of the NMDS analysis, control group in green, model group in blue; **(E)** Change in the gut flora comparing the two groups at the genus level.

A Venn diagram of the operational taxonomic units (OTUs) showed that the model group had the lowest number of unique OTUs (**Figure 3A**).

Effect of T2DM on the abundance of species in the gut microbiome: A principal component analysis (PCA) showed that the gut microbiome of diabetes mice was scattered not aggregated (**Figure 3B**). An unweighted principal coordinate analysis (PCoA) based on UniFrac algorithm showed significant difference between the model group and the control group (**Figure 3C**). NMDS (Nonmetric Multidimensional Scaling) is, like PCoA analysis, a ranking method based on a distance matrix of samples (of any type of distance). Unlike PCoA, NMDS is no longer based on the distance matrix values, but is a downscaling calculation based on the distance ranking order. NMDS showed significant difference between the model group and the control group (**Figure 3D**).

Effect of T2DM on the change in the species comprising the gut microbiome: At the genus level, the model group mice exhibited significant increased abundances of Helicobacter, Ruminococcaceae, Lachnospairceae, Cloatridiales, Bacteroides, and Ruminiclostridium and significantly decreased abundances of Muribaculaceae, and Lactobacillus compared with the control group (**Figure 3E**).

#### 3.2.3 Function prediction of predominant taxa

**Figure 4:**
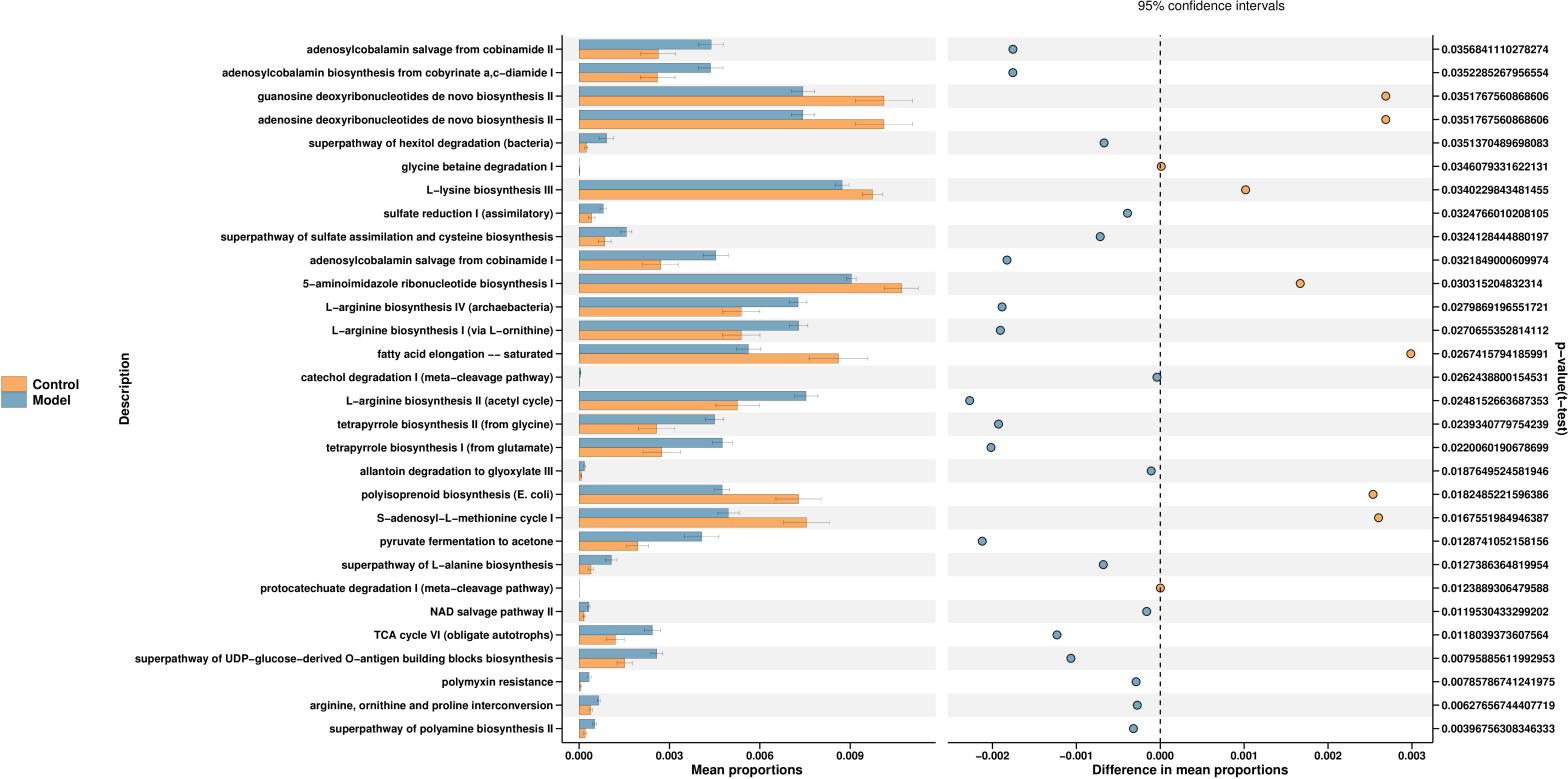
Function prediction of predominant taxa. PICRUSt2 is a software for estimating the functional capacity of microbial communities detected by 16s rDNA sequencing. The control group is shown in yellow and the model group in blue.

PICRUSt2 is a software to estimate the function capabilities of microbial communities identified in 16s DNA sequencing. According to a query of the KEGG database, fifty pathways enriched among the groups. The changes in different pathways are related to the L-lysine biosynthesis III, TCA cycle, polymyxin and others. We select the first 30 functions for picture display (**Figure 4**) (**Supplementary table S1**).

### 3.3 T2DM altered the urine metabolome

**Figure 5:**
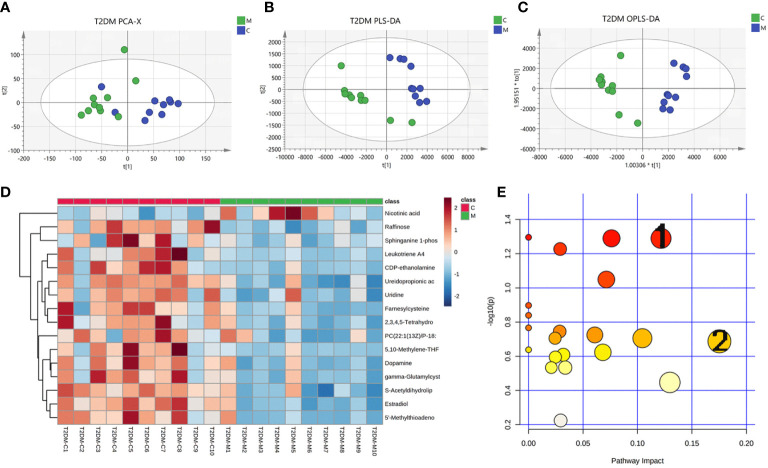
Changes in urinary metabolites in type 2 diabetic mice. Urine metabolome profiles were determined by LC-MS. **(A)** PCA analysis plot, control group in green, model group in blue; **(B)** PLS-DA analysis plot, control group in green, model group in blue; **(C)** OPLS-DA analysis plot, control group, blue for model group; **(D)** heat map of differential metabolites; **(E)** map of pathways, 1 for glycerophospholipid pathway and 2 for arachidonic acid pathway.

**Figure 6:**
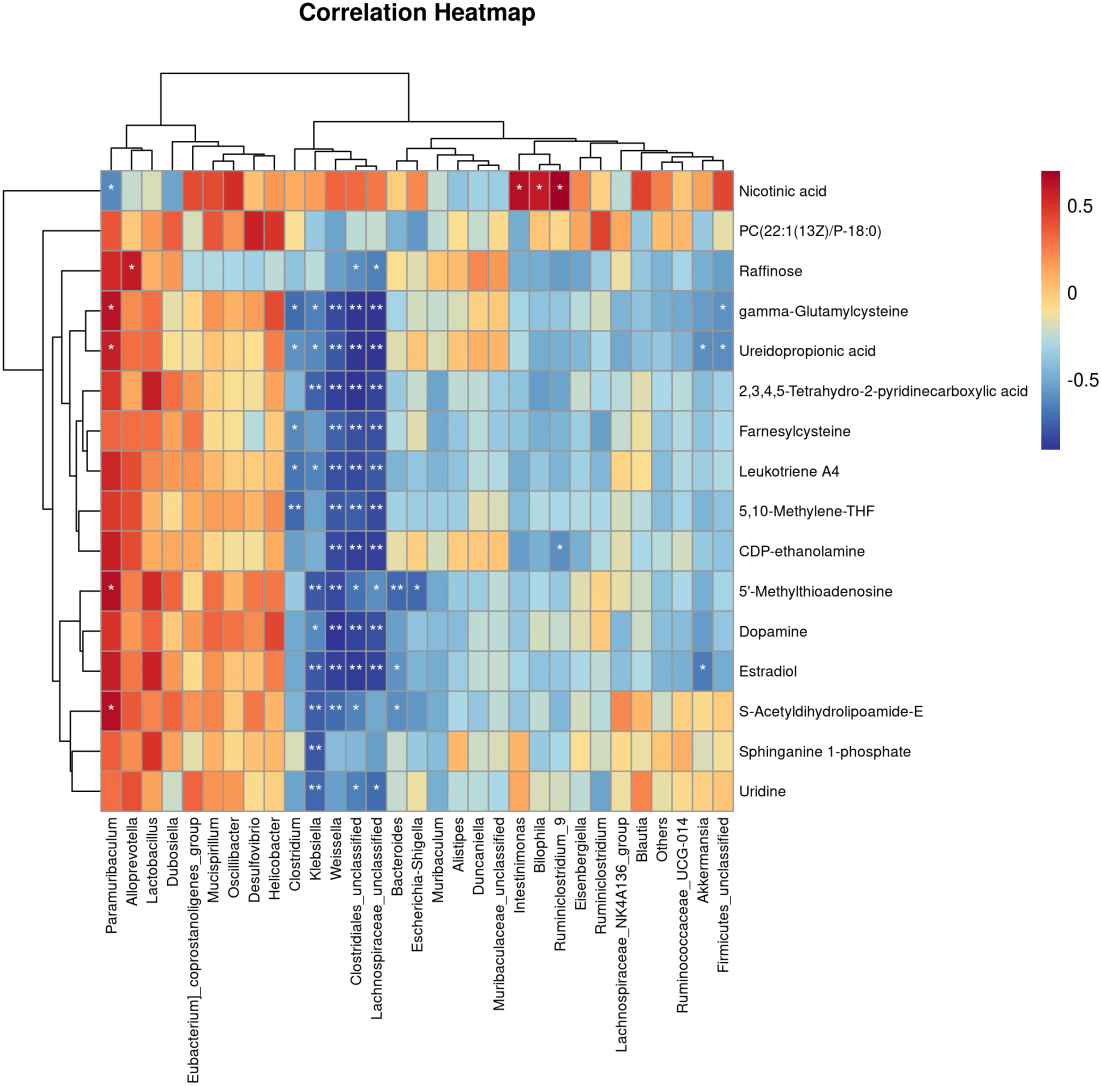
Correlations Between the Gut microbiota and metabolites in the T2DM mouse model. Pearman correlation analysis was used to further explore the correlation between the gut microbiome and the urinary metabolome. The horizontal coordinates are the gut flora and the vertical coordinates are the differential metabolites, where the redder the colour, the stronger the positive correlation, and the bluer the colour, the stronger the negative correlation. *p < 0.05, **p < 0.01.

The urine metabolome profiles were determined by LC-MS. The PCA plot and PLS-DA scores revealed that the metabolites of Model group were separated from those of Control group (**Figures 5A, B**). An OPLS-DA model was adopted to further analyze the differential metabolites between control and model group (**Figure 6C**).

Based on the results of OPLS-DA, we used the VIP as a threshold to further screen for differential metabolites between two groups. We further screened for VIP≥1.0, p-value < 0.05, and fold change≥ 2 or ≤ 0.5 metabolites as differential metabolites. 31 differential metabolites were detected in the control versus model group (**Figure 5D**) (**Table 1**). Urine differential metabolites.

**Table 1:** Urine differential metabolites.

number	tR (min)	Name	Molecular formula	Addition method	m/z measured value	m/z theoretical value	Error (ppm)	Trend	Multiplier	P	VIP
1	1.0804	Raffinose	C_18_H_32_O_16_	M+Na	527.1607	527.1588	3.604227037	↓**	0.327312219	8.70175E-05	1.43079
2	2.0118	4,6-Dihydroxyquinoline	C_9_H_7_NO_2_	M+H	162.0534	162.0555	-12.95852347	↓**	0.300581449	9.58041E-05	1.3803
3	2.6887	Metanephrine	C_10_H_15_NO_3_	M+Na	220.0915	220.095	-15.90222404	↓**	0.288269316	0.000251555	1.19564
4	2.69	Dopamine	C8H11NO2	M+Na	176.0689	176.0687	1.13592024	↓**	0.328582593	0.001235019	1.19564
5	2.7066	O-Phosphoethanolamine	C_2_H_8_NO_4_P	M+Na	164.012	164.0089	18.90141328	↓**	0.287697576	1.56862E-06	1.19564
6	3.1816	Cytidine	C_9_H_13_N_3_O_5_	M+H	244.0873	244.0933	-24.58076481	↓**	0.534099826	0.004046534	1.19564
7	3.3667	Octanoylglucuronide	C_14_H_24_O_8_	M+K	359.1156	359.1108	13.36634821	↓**	0.372185305	0.000444233	1.19564
8	3.3753	Pantetheine 4'-phosphate	C_11_H_23_N_2_O_7_PS	M+Na	381.0955	381.0861	24.66634181	↓**	0.520434545	0.003072497	1.19564
9	3.73	Leukotriene A4	C_20_H_30_O3	M+H	319.2281	319.2273	2.506051331	↓**	0.324647124	0.001654816	1.19564
10	4.3484	gamma-Glutamylcysteine	C_8_H_14_N_2_O_5_S	M+Na	273.0512	273.0521	-3.296074266	↑*	2.257294949	0.0130067	1.19564
11	4.6063	Farnesylcysteine	C_18_H_31_NO_2_S	M+Na	348.1961	348.1973	-3.446321956	↓**	0.199929665	0.000502727	1.19564
12	5.5635	9-cis-Retinoic acid	C_20_H_28_O_2_	M+K	339.1803	339.1726	22.70230555	↓**	0.102533232	0.000153634	1.18702
13	5.745	Phosphoserine	C_3_H_8_NO_6_P	M+H	186.0132	186.0167	-18.81551495	↓**	0.40818244	0.001002379	1.18702
14	5.7472	CDP-ethanolamine	C_11_H_20_N_4_O_11_P_2_	M+K	485.0283	485.0241	8.659363524	↓**	0.15066153	0.001893775	1.18702
15	5.8819	5-Methoxyindoleacetate	C_11_H_11_NO_3_	M+H	206.0764	206.0817	-25.71795555	↓**	0.228559091	0.000677246	1.18702
16	5.8851	Nicotinic acid	C_6_H_5_NO_2_	M+Na	146.0207	146.0218	-7.533121767	↓**	0.30034945	0.000861819	1.18702
17	6.4349	Estradiol	C_18_H_24_O_2_	M+H	273.1836	273.1855	-6.954981139	↓**	0.452411887	0.003926428	1.00002
18	6.5119	Dihydrofolic acid	C_19_H_21_N_7_O_6_	M+H	444.1697	444.1632	14.63426056	↓**	0.226021474	0.000465617	1.00002
19	6.6847	5,10-Methylene-THF	C_20_H_23_N_7_O_6_	M+H	458.1813	458.1788	5.456385149	↓**	0.165682053	0.001945198	1.00002
20	7.094	Ureidopropionic acid	C_4_H_8_N_2_O_3_	M+Na	155.0418	155.0433	-9.674716676	↓**	0.536622723	0.00011681	1.00002
21	7.2737	Tetrahydrodeoxycorticosterone	C_21_H_34_O_3_	M+K	373.1602	373.2145	-145.4927394	↓**	0.166738211	0.003191079	1.00002
22	7.3072	S-Acetyldihydrolipoamide-E	C_10_H_19_NO_2_S_2_	M+K	288.0513	288.0494	6.59609081	↓**	0.434940186	0.001089562	1.00002
23	7.3133	4-Trimethylammoniobutanoic acid	C_7_H_15_NO_2_	M+H	146.1151	146.1181	-20.53133732	↓**	0.183213763	1.85068E-05	1.00002
24	7.318	Uridine	C_9_H_12_N_2_O_6_	M+H	245.0736	245.0744	-3.264314837	↓**	0.240720577	4.96219E-06	1.00002
25	7.9493	2,3,4,5-Tetrahydro-2-pyridinecarboxylic acid	C_6_H_9_NO_2_	M+Na	150.0525	150.0531	-3.998584501	↓**	0.361945089	7.31985E-05	1.00002
26	7.967	2-Oxoarginine	C_6_H_11_N_3_O_3_	M+H	174.09	174.0879	12.06287169	↓**	0.231969556	0.000243991	1.00002
27	8.2491	3-Hydroxy-N6,N6,N6-trimethyl-L-lysine	C_9_H_21_N_2_O_3_	M+Na	227.1438	228.145	-4388.437178	↓**	0.191678219	0.002720685	1.00002
28	10.2327	5'-Methylthioadenosine	C_11_H_15_N_5_O_3_S	M+H	298.0961	298.0974	-4.360990737	↓**	0.283002531	0.000276381	1.40139
29	10.3631	Carbamoyl phosphate	CH_4_NO_5_P	M+H	141.9877	141.9905	-19.71962913	↓**	0.531233198	0.003446439	1.40139
30	10.7896	PC(22:1(13Z)/P-18:0)	C_48_H_94_NO_7_P	M+Na	850.6664	850.6666	-0.235109736	↓*	0.446311894	0.017805383	1.40139
31	12.156	Sphinganine 1-phosphate	C_18_H_40_NO_5_P	M+H	382.2754	382.2722	8.370998467	↓*	0.392632102	0.01545641	1.3803

Finally, we used KEGG database to identify the metabolic pathways. The results showed that the differential metabolites participated in metabolic pathways involving Pyruvate metabolic pathway, Glycerophospholipid metabolic pathway, Arachidonic acid metabolic pathway, and Glutathione metabolism, suggesting that the distance of many pathways was implicated in disorders of glycolipid metabolism (**Figure 5E**) (**Supplementary table S2**).

### 3.4 Correlations between the gut microbiota and metabolites in the T2DM mouse model

**Figure 7:**
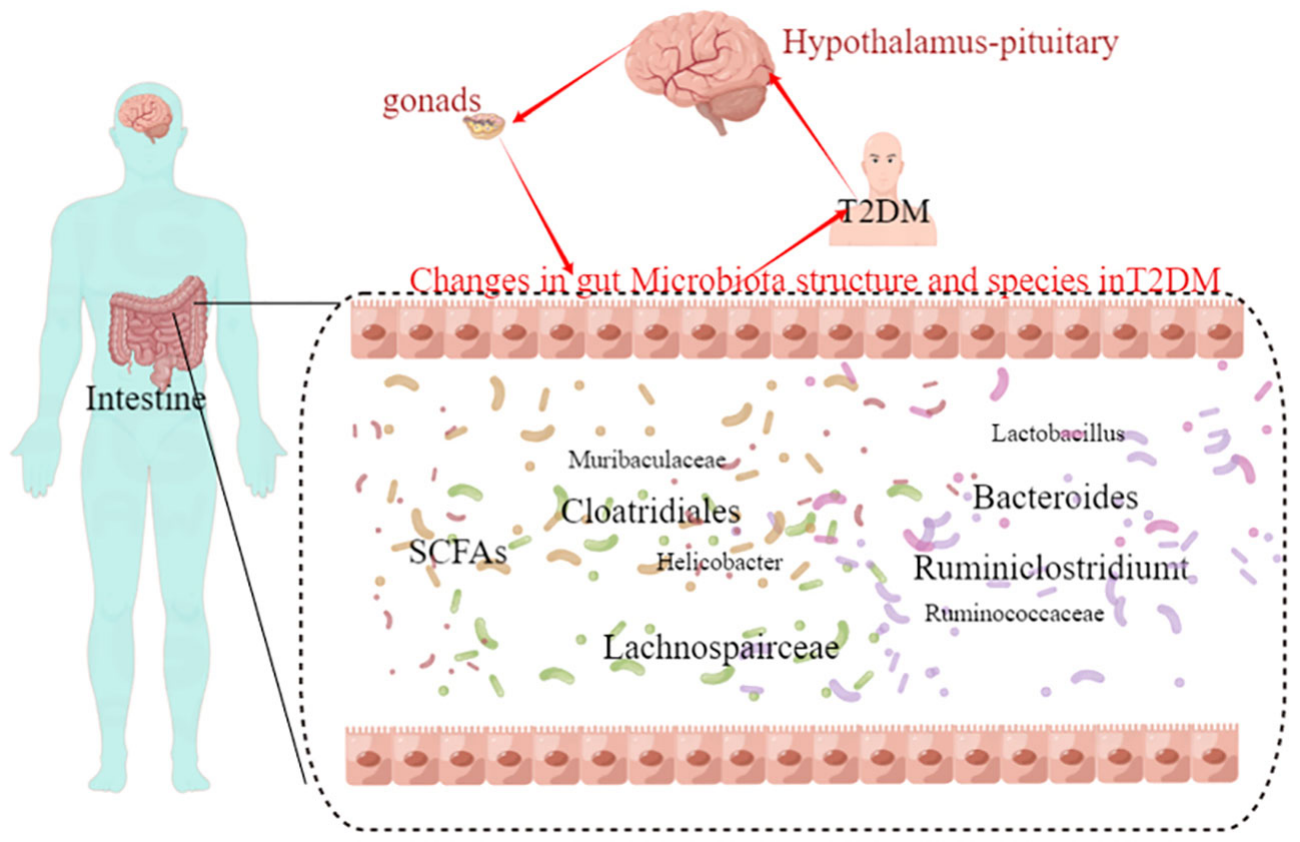
The hypothalamic-pituitary-gonadal axis-gut microbiota may be a novel direction underlying T2DM.

Pearman correlation analysis was used to further explore the correlation between the gut microbiome and the urine metabolome. The Pearman correlation coefficients between the differential metabolites and the top 30 differential microbes were calculated. The results showed that there was a significant correlation between Weissella, Klebsiella, Clostridiales, Lachnospiraceae and uridine, sphinganie 1-phosphate, S-Acetyldihydrolipoamide-E, Estradiol, Dopamine (**Figure 7**). Pearman correlation analysis showed a strong correlation between dopamine and gonadal, estradiol and gut microbiota, may be a novel direction underlying T2DM. (**Figure 7**).

## 4 Discussion

T2DM is the most serious metabolic disease in the world, long-term disorders of blood glucose metabolism can cause vascular endothelial damage, which leads to functional and structural damage or even failure of heart, brain, kidney, retina, large and small blood vessels and other tissues and organs, but its pathogenesis is still unclear, but studies have shown that the onset of T2DM is related to intestinal flora and urinary metabolites, but the relationship between the two is unclear, so this experiment By establishing a mouse T2DM model, By establishing a mouse T2DM model, applying 16s rDNA sequencing technology to detect changes in the structure and species and amount of intestinal flora in mice, and detecting changes in urinary metabolites in type 2 diabetic mice by UPLC-Q-TOF-MS technology, analyzing the differential metabolites and constructing differential metabolic pathways, and finally applying Pearman correlation analysis to explore the relationship between intestinal flora in type 2 diabetic mice.

In this experiment, we used 16s rDNA sequencing technology to detect changes in the structure, species and abundance of the intestinal flora in mice, and analyzed the differences between the intestinal flora of type 2 diabetic mice and untreated mice by beta diversity. The results showed that Lactobacillus and Helicobacter pylori are closely associated with T2DM. Lactobacillus is a group of gram-positive bacteria found mainly in the human oral cavity, intestinal tract, reproductive system and urinary system. The results of this study showed that the number of Lactobacillus was significantly reduced in type 2 diabetic mice. Lactobacillus can improve insulin resistance, lower blood glucose levels and reduce the inflammatory response associated with diabetes (Yan et al., 2019; Wang et al., 2023). First, Lactobacillus may prevent T2DM by improving intestinal barrier function. Intestinal barrier function refers to the integrity and functional state of the intestinal mucosal layer and is essential for maintaining the balance of the intestinal microbial community. Studies have shown that Lactobacillus can improve the integrity of the intestinal barrier and mucus secretion, reduce the invasion of harmful microorganisms and the inflammatory response, and thus prevent the development of T2DM (Yan et al., 2019; Eliuz Tipici et al., 2023). Secondly, Lactobacillus can treat T2DM by improving insulin resistance. Insulin resistance is one of the main pathophysiological underpinnings of T2DM and refers to the reduced response of the body’s cells to insulin, leading to an increase in blood glucose levels (Tang et al., 2023). Studies have shown that Lactobacillus may be useful in the treatment of T2DM by increasing the production of short-chain fatty acids, modulating the immune system and metabolic pathways, reducing the inflammatory response and improving insulin resistance (Gu et al., 2023). Dietary SCFA supplementation prevented and reversed high-fat diet-induced metabolic abnormalities in mice by reducing PPARγ expression and activity. This increased the expression of mitochondrial uncoupling protein 2 and increased the ratio of AMP to ATP, thereby stimulating oxidative metabolism in liver and adipose tissue via AMPK (den Besten et al., 2015).

Helicobacter pylori (H. pylori) is a bacterium that can live in the stomach and has been linked to many digestive disorders, including gastritis, stomach ulcers and stomach cancer. In recent years, studies have shown that H. pylori is also associated with T2DM. A clinical study showed that Helicobacter pylori infection associated with risk of type 2 diabetes in middle-aged and elderly Chinese (Han et al., 2016; Zhou et al., 2022). It has also been suggested that eradicating H. pylori from the body may be an important treatment for type 2 diabetes (Shi et al., 2018; Shi and Chen, 2021). Firstly, H. pylori infection can lead to chronic inflammation of the stomach lining and abnormal secretion of gastric acid, which can affect the digestion of food and absorption of nutrients, which in turn affects metabolism and insulin secretion (Pachathundikandi et al., 2023; Yu et al., 2023). Secondly, H. pylori infection can also affect the internal environment of the gastrointestinal tract and promote chronic low-grade inflammation, thereby inducing an inflammatory and immune response in the body, which further affects insulin sensitivity (Azami et al., 2021). In addition, H. pylori directly damage pancreatic beta cells and interfere with insulin synthesis and secretion, ultimately leading to the development of T2DM (So et al., 2009). Some studies have also found that H. pylori infection is closely associated with conditions such as obesity and metabolic syndrome, which are also risk factors for T2DM (Lim et al., 2019). Therefore, H. pylori infection may influence the development and progression of T2DM through multiple pathways. In conclusion, intestinal microorganisms such as Lactobacillus and Helicobacter pylori are closely associated with T2DM, and these microorganisms may be involved in physiological processes such as insulin resistance, blood glucose regulation and inflammatory responses through a variety of pathways; therefore, by studying changes in the species and numbers of intestinal flora in T2DM, new therapeutic targets may be identified.

Through PICRUSt2, we have established a mapping between flora and function. The experimental results show that the arginine biosynthesis pathway and the tricarboxylic acid cycle pathway are closely linked to T2DM.

Arginine is a very important amino acid in the body, and studies have shown that the arginine biosynthesis pathway is closely linked to type 2 diabetes. Arginine-stimulated insulin secretion provides a clinical measure of beta cell functional mass and secretory capacity (Hannon et al., 2018). Arginine supplementation improves insulin sensitivity in healthy people, obese people, type 2 diabetes and coronary artery disease. Insufficient insulin secretion and/or utilisation is a major cause of T2DM and the insulin signalling pathway is the main pathway by which insulin acts on target cells to produce biological effects, with the insulin receptor (InsR) and the insulin-like growth factor receptor (IGF-1R) being important components of the insulin signalling pathway. improve vascular function and is associated with reduced Nox4-dependent oxygen radical production by upregulating the IRS-1/AKT/NOS/NO signalling pathway (Federici et al., 2004). Second, Arginine also plays an important role in the inflammatory response and oxidative stress, which are among the most important factors in the development and progression of T2DM. Arginine can reduce the release and expression of inflammatory factors by inhibiting the activation of the NF-κB pathway, a transcription factor that regulates the expression of a variety of inflammatory factors, including IL-6 and TNF-α, to reduce leukocyte infiltration and tissue damage (Liang et al., 2022), thereby ameliorating insulin resistance and abnormal glucose metabolism. In addition, arginine can promote the synthesis and activity of antioxidants such as SOD and inhibit the production and accumulation of ROS, thereby protecting cells from oxidative stress (Ginovyan et al., 2023). In addition, arginine can regulate glucolipid metabolism in diabetic rats, increase insulin sensitivity, improve insulin resistance, reduce plasma lipid levels and increase the body’s antioxidant capacity to reduce the metabolic diseases caused by type 2 diabetes (Kurek et al., 2022). In conclusion, arginine plays an important role in insulin secretion, inflammatory response and oxidative stress and genes in the arginine synthesis pathway may be closely associated with the development and progression of T2DM. Therefore, arginine synthesis and metabolism may become an important target in the treatment of T2DM.

The tricarboxylic acid (TCA) cycle is an important intracellular metabolic pathway involved in cellular energy metabolism and biosynthetic reactions. T2DM is a metabolic disease whose onset and progression are associated with multiple metabolic abnormalities. A number of studies have suggested a possible link between the TCA cycle and T2DM. Firstly, the TCA cycle is one of the main pathways for the production of ATP. Succinate dehydrogenase (SDH) is one of five mitochondrial complexes involved in the electron transport chain and also plays a role in the tricarboxylic acid (TCA) cycle by catalysing the oxidation of succinate to fumarate, suggesting that SDH deficiency is a pathogenic driver of β-cell metabolic dysregulation and mitochondrial dysfunction (Lee et al., 2022). Another study showed that inhibition of SDH significantly impairs the efficiency of glucose-hyperpolarised beta-cell mitochondria and that the resulting reduction in ATP synthesis disrupts the SSC, thereby reducing the insulin beta-cell stimulation-secretion coupling (SSC) (Edalat et al., 2015). Secondly, in people with diabetes, there is insulin resistance, which means that the effects of insulin on tissues are reduced, making it difficult for cells to use glucose efficiently, which can lead to a reduction in intracellular ATP production and thus affect normal metabolic and biosynthetic reactions. In addition, oxidative stress and inflammatory responses play an important role in the development and progression of T2DM. There are several key enzymes and mediators in the tricarboxylic acid cycle pathway that are closely related to oxidative stress and inflammatory responses (He et al., 2023; Liu et al., 2023). In conclusion, there is a strong relationship between the tricarboxylic acid cycle and T2DM. The tricarboxylic acid cycle is involved in various physiological and pathological processes, such as energy metabolism, metabolic function, oxidative stress and inflammatory response, and plays an important role in the onset and development of T2DM. Therefore, further research and exploration of the relationship between the tricarboxylic acid cycle and T2DM is of great importance in elucidating the mechanisms of T2DM pathogenesis and exploring new therapeutic targets.

In this study, urinary metabolites of mice were detected by UPLC-Q-TOF-MS, and the results showed that there were differences in urinary metabolites in type 2 diabetic mice compared with untreated mice. 31 differential markers were identified by OPLS-DA to further investigate the differential metabolites between the two groups, which may be related to the glycerophospholipid pathway and the arachidonic acid pathway.

A total of 31 differential metabolites were analysed and, in addition to amino acids, we found that levels of the hormones dopamine and estradiol were significantly reduced in type 2 diabetic mice. Dopamine is a neurotransmitter secreted by the hypothalamus, is commonly used to treat neurological conditions such as Parkinson’s disease, but previous studies have shown a link between dopamine levels and type 2 diabetes (Lopez Vicchi et al., 2016). A study shows that the antipsychotic dopamine 2 receptor antagonist diphenylbutylpiperidine improves blood glucose in experimental obesity by inhibiting succinyl coenzyme A:3-ketoacyl coenzyme A transferase (Tabatabaei Dakhili et al., 2023). Another study showed that dopamine acts directly on insulin-sensitive tissues *via* several receptors to regulate glucose uptake and insulin receptor phosphorylation and to regulate metabolic functions, adenosine monophosphate kinase (AMPK) phosphorylation in liver, adipose tissue and skeletal muscle, and lipid metabolism-related proteins in adipose tissue (Tavares et al., 2021). In addition, we detected a decrease in estradiol levels in the urinary metabolites of type 2 diabetic mice. Estradiol is a steroid hormone secreted by the gonadal organs. Studies have shown that lipocalin and insulin resistance form a vicious circle that exacerbates type 2 diabetes, and that modulation of estrogen receptors by diet or pharmacological intervention can improve lipocalin resistance and thus insulin resistance in type 2 diabetes (Chattopadhyay et al., 2022). Sex hormone-binding globulin prevents insulin resistance to endoplasmic reticulum stress in hepatocytes, reducing type 2 diabetes and counteracting the development of obesity caused by high-fat diets, and sex hormone-binding globulin improves insulin sensitivity by stimulating hepatocytes (Bourebaba et al., 2022).

Disturbances in glycerophospholipid (GPL) metabolism may play a role in the pathogenesis of T2DM. Studies have shown that changes in the levels of GPLs are associated with insulin resistance and T2DM (Lu et al., 2016). Gro3P phosphatase was found to regulate glycolysis and glucose oxidation, cellular redox and ATP production, gluconeogenesis, glycerolipid synthesis and fatty acid oxidation in pancreatic β-cells and hepatocytes through the control of glycerol-3-phosphate (Gro3P) levels, and glucose stimulates insulin secretion and the response to β-cell metabolic stress (Mugabo et al., 2016). In addition, inflammation and oxidative stress are thought to be important in the pathogenesis of T2DM, and studies have shown that lysophosphatidylinositol (LPI) induces cells to secrete IL-6 and TNF-α, leading to an inflammatory response in the body (Kurano et al., 2021). Another study showed that sphingosine 1-phosphate (S1P) is an important lipid molecule and that S1P reduces mitochondrial ROS production, leading to upregulation of insulin signalling and glucose uptake in insulin-induced IR (Fang et al., 2020). Furthermore, dysregulation of key enzymes involved in GPL synthesis and degradation, such as phosphocholine cytidylyltransferase (CCT) and phospholipase A2 has been associated with insulin resistance and T2DM (Sakai et al., 2014; Kuefner, 2021; Siddiqui et al., 2022). Meanwhile, triglyceride levels are also higher in diabetics, which may be related to the GPL pathway. In addition, the GPL pathway may also influence insulin resistance and insulin secretion by regulating fatty acid synthesis and metabolism, thereby affecting the regulation of blood glucose levels (Lee and Ridgway, 2020).

Arachidonic acid (AA) is a 20-carbon polyunsaturated fatty acid found in phospholipids in the plasma membrane. The three main pathways of AA metabolism are mediated by cyclooxygenase enzymes, lipoxygenase enzymes and cytochrome P450 enzymes. Studies have shown that abnormalities in arachidonic acid metabolism may be associated with the development and progression of type 2 diabetes. Firstly, abnormal arachidonic acid metabolism may lead to insulin resistance (Mak et al., 2020; Isse et al., 2022). Study has shown that arachidonic acid and its metabolites affect the function and amount of insulin secreted by pancreatic beta cells (Bosma et al., 2022). Secondly, AA metabolic abnormalities may also be related to inflammation and abnormal insulin secretion (Rengachar et al., 2022). PGE2 plays an important regulatory role in insulin secretion, and its decreased level may lead to abnormal insulin secretion and elevated blood glucose levels (Wang et al., 2022). Arachidonic acid (AA)-rich ARASCO oil reduces hyperglycaemia, restores insulin sensitivity, inhibits inflammation, increases plasma lipoxin A4 levels and restores the altered antioxidant status of HFD + STZ-induced diabetic animals to near normal (Gundala and Das, 2019; Zhong et al., 2022).

Finally, the relationship between gut flora species and urinary metabolites in type 2 diabetic mice was investigated using Pearman correlation analysis, with the aim of integrating the changes in gut flora and urinary metabolites in T2DM, analysing the possible mechanisms of T2DM and providing new ideas for clinical drug development. The results showed that there were significant correlations between Klebsiella, Clostridium and Lachnospira and uridine, sphingosine-1-phosphate, S-acetylhydantoin-E, estradiol and dopamine. We therefore propose the hypothalamic-pituitary-gonadal axis microbes as a new mechanism for the treatment of type 2 diabetes. Both clinical studies and basic research have shown that type 2 diabetes leads to dysfunction of the hypothalamic-pituitary-gonadal axis, which affects hormone secretion and causes histological damage to the testes (George et al., 2010; Barsiah et al., 2019; Dudek et al., 2019). Tenofovir disoproxil fumarate-loaded silver nanoparticles have been shown to restore hypothalamic-pituitary-gonadal axis function, normalise hormonal profiles and improve testicular function and structure to alleviate reproductive dysfunction in diabetic rats (Olojede et al., 2022). Evidence that the variety and diversity of the gut flora is regulated by oestrogen (Acharya et al., 2023; Song et al., 2023). Whereas changes in the type and number of intestinal flora are present in both type 2 diabetics and animals, and the application of probiotics to regulate intestinal flora is also a means of treating type 2 diabetes, therefore, we propose pituitary gonadal axis-microbes as a new strategy for the direction of type 2 diabetes. However, Urinary dopamine cannot be considered as the marker of hypothalamic dopamine production. Dopamine is produced by many neurons in the CNS and is quickly metabolized. In addition, dopamine is produced by peripheral tissues including the kidney. All in all a large number of experiments will be needed at a later stage to validate this part.

In conclusion, by establishing a type 2 diabetic mouse model and studying changes in its gut flora and urinary metabolites, our experimental results showed that the type and number of gut flora were altered in type 2 diabetic mice, with significantly lower levels of beneficial bacteria such as lactobacilli and significantly higher levels of harmful bacteria such as Helicobacter pylori. At the metabolic level, 31 different metabolites were examined, with significantly lower levels of estradiol and dopamine, which may be related to the glycerophospholipid and arachidonic acid pathways. Pearman correlation analysis showed a strong correlation between dopamine and gonadal, estradiol and gut microbiota, may be a novel direction underlying T2DM, the aim is to provide new ideas for clinical treatment and basic research.

## 5 Limitations

Although we have elucidated the changes in the structure and quantity of gut flora and urinary metabolites in type 2 diabetic mice, we have not shown through experimental or clinical studies how gut flora and urinary metabolites regulate T2DM, which is one of the elements that needs to be further investigated in the future. Secondly, there are many changes in gut flora and urinary metabolites in type 2 diabetic mice, and we have only focused on a few that are significantly different. It is possible that others are also important target mechanisms that affect T2DM, and more analysis and research is needed to complement the possible targets of T2DM in order to provide a more adequate basic experimental basis for the clinical development and application of drugs to treat T2DM. Finally, although we have proposed the pituitary gonadal axis-microbes as a new strategy for the direction of T2DM, we have not tested this hypothesis and subsequent experiments are needed to verify this part.

## Data availability statement

The raw data supporting the conclusions of this article will be made available by the authors, without undue reservation.

## Ethics statement

The animal study was reviewed and approved by the Medical Ethics Committee of Tianjin University of Traditional Chinese Medicine.

## Author contributions

ZS: Conceived and designed the experiments; Performed the experiments; Analyzed and interpreted the data; Wrote the paper. AY: Conceived and designed the experiments; Performed the experiments; Wrote the paper. ZG: Performed the experiments; Analyzed and interpreted the data. YZ: Performed the experiments; Analyzed and interpreted the data. TW: Performed the experiments; Analyzed and interpreted the data. ZL: Performed the experiments; Analyzed and interpreted the data. ZY: Analyzed and interpreted the data; Wrote the paper. RC: Performed the experiments; Analyzed and interpreted the data; YW: Conceived and designed the experiments; Contributed reagents, materials, analysis tools or data; Wrote the paper. All authors contributed to the article and approved the submitted version.
