# Supplementary material for: Targeting metabolic pathways: a novel therapeutic direction for type 2 diabetes
Source: Front Cell Infect Microbiol. 2023 Aug 2;13:1218326. doi: 10.3389/fcimb.2023.1218326 (PMC10433779; doi:10.3389/fcimb.2023.1218326)
Supplement: Supplementary file 1 [file Table_1.docx]

**Supplementary Table 1 Functional pathways of the intestinal flora**

| **pathways description** | **p_value** |
| --- | --- |
| 1,4-dihydroxy-2-naphthoate biosynthesis I | 0.04 |
| 5-aminoimidazole ribonucleotide biosynthesis I | 0.03 |
| adenosine deoxyribonucleotides de novo biosynthesis II | 0.04 |
| adenosylcobalamin biosynthesis from cobyrinate a,c-diamide I | 0.04 |
| adenosylcobalamin salvage from cobinamide I | 0.03 |
| adenosylcobalamin salvage from cobinamide II | 0.04 |
| allantoin degradation to glyoxylate III | 0.02 |
| arginine, ornithine and proline interconversion | 0.01 |
| Calvin-Benson-Bassham cycle | 0.05 |
| catechol degradation I (meta-cleavage pathway) | 0.03 |
| dTDP-N-acetylthomosamine biosynthesis | 0.04 |
| fatty acid elongation -- saturated | 0.03 |
| formaldehyde assimilation I (serine pathway) | 0.05 |
| glycine betaine degradation I | 0.03 |
| guanosine deoxyribonucleotides de novo biosynthesis II | 0.04 |
| L-arginine biosynthesis I (via L-ornithine) | 0.03 |
| L-arginine biosynthesis II (acetyl cycle) | 0.02 |
| L-arginine biosynthesis IV (archaebacteria) | 0.03 |
| L-lysine biosynthesis III | 0.03 |
| L-ornithine biosynthesis | 0.04 |
| NAD salvage pathway II | 0.01 |
| nitrate reduction I (denitrification) | 0.04 |
| peptidoglycan biosynthesis V (&beta;-lactam resistance) | 0.04 |
| polyisoprenoid biosynthesis (E. coli) | 0.02 |
| polymyxin resistance | 0.01 |
| protocatechuate degradation I (meta-cleavage pathway) | 0.01 |
| pyruvate fermentation to acetone | 0.01 |
| pyruvate fermentation to isobutanol (engineered) | 0.04 |
| S-adenosyl-L-methionine cycle I | 0.02 |
| sulfate reduction I (assimilatory) | 0.03 |
| superpathway of adenosine nucleotides de novo biosynthesis II | 0.04 |
| superpathway of demethylmenaquinol-8 biosynthesis | 0.05 |
| superpathway of hexitol degradation (bacteria) | 0.04 |
| superpathway of L-alanine biosynthesis | 0.01 |
| superpathway of L-threonine biosynthesis | 0.04 |
| superpathway of menaquinol-11 biosynthesis | 0.05 |
| superpathway of menaquinol-12 biosynthesis | 0.05 |
| superpathway of menaquinol-13 biosynthesis | 0.05 |
| superpathway of menaquinol-7 biosynthesis | 0.05 |
| superpathway of menaquinol-8 biosynthesis I | 0.05 |
| superpathway of phylloquinol biosynthesis | 0.04 |
| superpathway of polyamine biosynthesis II | 0.00 |
| superpathway of pyrimidine deoxyribonucleoside salvage | 0.05 |
| superpathway of sulfate assimilation and cysteine biosynthesis | 0.03 |
| superpathway of UDP-glucose-derived O-antigen building blocks biosynthesis | 0.01 |
| TCA cycle VI (obligate autotrophs) | 0.01 |
| tetrapyrrole biosynthesis I (from glutamate) | 0.02 |
| tetrapyrrole biosynthesis II (from glycine) | 0.02 |
| toluene degradation I (aerobic) (via o-cresol) | 0.04 |
| toluene degradation II (aerobic) (via 4-methylcatechol) | 0.04 |
