# Supplementary material for: Targeting metabolic pathways: a novel therapeutic direction for type 2 diabetes
Source: Front Cell Infect Microbiol. 2023 Aug 2;13:1218326. doi: 10.3389/fcimb.2023.1218326 (PMC10433779; doi:10.3389/fcimb.2023.1218326)
Supplement: Supplementary file 2 [file Table_2.docx]

**Supplementary Table 2 Urine differential metabolites**

| number | tR（min） | Name | Molecular formula | Addition method | m/z measured value | m/z theoretical value | Error (ppm) | Trend | Multiplier | P | VIP |
| --- | --- | --- | --- | --- | --- | --- | --- | --- | --- | --- | --- |
| 1 | 1.0804 | Raffinose | C18H32O16 | M+Na | 527.1607 | 527.1588 | 3.604227037 | ↓** | 0.327312219 | 8.70175E-05 | 1.43079 |
| 2 | 2.0118 | 4,6-Dihydroxyquinoline | C9H7NO2 | M+H | 162.0534 | 162.0555 | -12.95852347 | ↓** | 0.300581449 | 9.58041E-05 | 1.3803 |
| 3 | 2.6887 | Metanephrine | C10H15NO3 | M+Na | 220.0915 | 220.095 | -15.90222404 | ↓** | 0.288269316 | 0.000251555 | 1.19564 |
| 4 | 2.69 | Dopamine | C8H11NO2 | M+Na | 176.0689 | 176.0687 | 1.13592024 | ↓** | 0.328582593 | 0.001235019 | 1.19564 |
| 5 | 2.7066 | O-Phosphoethanolamine | C2H8NO4P | M+Na | 164.012 | 164.0089 | 18.90141328 | ↓** | 0.287697576 | 1.56862E-06 | 1.19564 |
| 6 | 3.1816 | Cytidine | C9H13N3O5 | M+H | 244.0873 | 244.0933 | -24.58076481 | ↓** | 0.534099826 | 0.004046534 | 1.19564 |
| 7 | 3.3667 | Octanoylglucuronide | C14H24O8 | M+K | 359.1156 | 359.1108 | 13.36634821 | ↓** | 0.372185305 | 0.000444233 | 1.19564 |
| 8 | 3.3753 | Pantetheine 4'-phosphate | C11H23N2O7PS | M+Na | 381.0955 | 381.0861 | 24.66634181 | ↓** | 0.520434545 | 0.003072497 | 1.19564 |
| 9 | 3.73 | Leukotriene A4 | C20H30O3 | M+H | 319.2281 | 319.2273 | 2.506051331 | ↓** | 0.324647124 | 0.001654816 | 1.19564 |
| 10 | 4.3484 | gamma-Glutamylcysteine | C8H14N2O5S | M+Na | 273.0512 | 273.0521 | -3.296074266 | ↑* | 2.257294949 | 0.0130067 | 1.19564 |
| 11 | 4.6063 | Farnesylcysteine | C18H31NO2S | M+Na | 348.1961 | 348.1973 | -3.446321956 | ↓** | 0.199929665 | 0.000502727 | 1.19564 |
| 12 | 5.5635 | 9-cis-Retinoic acid | C20H28O2 | M+K | 339.1803 | 339.1726 | 22.70230555 | ↓** | 0.102533232 | 0.000153634 | 1.18702 |
| 13 | 5.745 | Phosphoserine | C3H8NO6P | M+H | 186.0132 | 186.0167 | -18.81551495 | ↓** | 0.40818244 | 0.001002379 | 1.18702 |
| 14 | 5.7472 | CDP-ethanolamine | C11H20N4O11P2 | M+K | 485.0283 | 485.0241 | 8.659363524 | ↓** | 0.15066153 | 0.001893775 | 1.18702 |
| 15 | 5.8819 | 5-Methoxyindoleacetate | C11H11NO3 | M+H | 206.0764 | 206.0817 | -25.71795555 | ↓** | 0.228559091 | 0.000677246 | 1.18702 |
| 16 | 5.8851 | Nicotinic acid | C6H5NO2 | M+Na | 146.0207 | 146.0218 | -7.533121767 | ↓** | 0.30034945 | 0.000861819 | 1.18702 |
| 17 | 6.4349 | Estradiol | C18H24O2 | M+H | 273.1836 | 273.1855 | -6.954981139 | ↓** | 0.452411887 | 0.003926428 | 1.00002 |
| 18 | 6.5119 | Dihydrofolic acid | C19H21N7O6 | M+H | 444.1697 | 444.1632 | 14.63426056 | ↓** | 0.226021474 | 0.000465617 | 1.00002 |
| 19 | 6.6847 | 5,10-Methylene-THF | C20H23N7O6 | M+H | 458.1813 | 458.1788 | 5.456385149 | ↓** | 0.165682053 | 0.001945198 | 1.00002 |
| 20 | 7.094 | Ureidopropionic acid | C4H8N2O3 | M+Na | 155.0418 | 155.0433 | -9.674716676 | ↓** | 0.536622723 | 0.00011681 | 1.00002 |
| 21 | 7.2737 | Tetrahydrodeoxycorticosterone | C21H34O3 | M+K | 373.1602 | 373.2145 | -145.4927394 | ↓** | 0.166738211 | 0.003191079 | 1.00002 |
| 22 | 7.3072 | S-Acetyldihydrolipoamide-E | C10H19NO2S2 | M+K | 288.0513 | 288.0494 | 6.59609081 | ↓** | 0.434940186 | 0.001089562 | 1.00002 |
| 23 | 7.3133 | 4-Trimethylammoniobutanoic acid | C7H15NO2 | M+H | 146.1151 | 146.1181 | -20.53133732 | ↓** | 0.183213763 | 1.85068E-05 | 1.00002 |
| 24 | 7.318 | Uridine | C9H12N2O6 | M+H | 245.0736 | 245.0744 | -3.264314837 | ↓** | 0.240720577 | 4.96219E-06 | 1.00002 |
| 25 | 7.9493 | 2,3,4,5-Tetrahydro-2-pyridinecarboxylic acid | C6H9NO2 | M+Na | 150.0525 | 150.0531 | -3.998584501 | ↓** | 0.361945089 | 7.31985E-05 | 1.00002 |
| 26 | 7.967 | 2-Oxoarginine | C6H11N3O3 | M+H | 174.09 | 174.0879 | 12.06287169 | ↓** | 0.231969556 | 0.000243991 | 1.00002 |
| 27 | 8.2491 | 3-Hydroxy-N6,N6,N6-trimethyl-L-lysine | C9H21N2O3 | M+Na | 227.1438 | 228.145 | -4388.437178 | ↓** | 0.191678219 | 0.002720685 | 1.00002 |
| 28 | 10.2327 | 5'-Methylthioadenosine | C11H15N5O3S | M+H | 298.0961 | 298.0974 | -4.360990737 | ↓** | 0.283002531 | 0.000276381 | 1.40139 |
| 29 | 10.3631 | Carbamoyl phosphate | CH4NO5P | M+H | 141.9877 | 141.9905 | -19.71962913 | ↓** | 0.531233198 | 0.003446439 | 1.40139 |
| 30 | 10.7896 | PC(22:1(13Z)/P-18:0) | C48H94NO7P | M+Na | 850.6664 | 850.6666 | -0.235109736 | ↓* | 0.446311894 | 0.017805383 | 1.40139 |
| 31 | 12.156 | Sphinganine 1-phosphate | C18H40NO5P | M+H | 382.2754 | 382.2722 | 8.370998467 | ↓* | 0.392632102 | 0.01545641 | 1.3803 |
