# Supplementary material for: Targeting metabolic pathways: a novel therapeutic direction for type 2 diabetes
Source: Front Cell Infect Microbiol. 2023 Aug 2;13:1218326. doi: 10.3389/fcimb.2023.1218326 (PMC10433779; doi:10.3389/fcimb.2023.1218326)
Supplement: Supplementary file 3 [file Table_3.docx]

**Supplementary Table 3 Urinary metabolic pathways**

| **Pathway Name** | **p_value** |
| --- | --- |
| Pyrimidine metabolism | 0.0066066 |
| Lysine degradation | 0.012373 |
| One carbon pool by folate | 0.012774 |
| Glycerophospholipid metabolism | 0.033111 |
| Pantothenate and CoA biosynthesis | 0.053599 |
| Sphingolipid metabolism | 0.064222 |
| D-Arginine and D-ornithine metabolism | 0.077705 |
| Linoleic acid metabolism | 0.096198 |
| Nitrogen metabolism | 0.11433 |
| Cysteine and methionine metabolism | 0.13948 |
| Glycine, serine and threonine metabolism | 0.13948 |
| Arachidonic acid metabolism | 0.1605 |
| Tryptophan metabolism | 0.19673 |
| Tyrosine metabolism | 0.20411 |
| alpha-Linolenic acid metabolism | 0.23177 |
| Arginine biosynthesis | 0.24727 |
| Nicotinate and nicotinamide metabolism | 0.26246 |
| Retinol metabolism | 0.29196 |
| Terpenoid backbone biosynthesis | 0.30627 |
| Pentose and glucuronate interconversions | 0.30627 |
| Citrate cycle (TCA cycle) | 0.33407 |
| beta-Alanine metabolism | 0.34757 |
| Pyruvate metabolism | 0.36079 |
| Glycolysis / Gluconeogenesis | 0.41116 |
| Folate biosynthesis | 0.42314 |
| Galactose metabolism | 0.42314 |
| Alanine, aspartate and glutamate metabolism | 0.43488 |
| Glutathione metabolism | 0.43488 |
| Steroid hormone biosynthesis | 0.51498 |
